# A Dual-Modal Biosensing Approach to Evaluate Cortico-Muscular Coupling in Adolescent Idiopathic Scoliosis

**DOI:** 10.3390/bios16080451

**Published:** 2026-08-20

**Authors:** Chen Liu, Bolin Mai, Kaiqi Wang, Xiaomin Chen, Yinling Sun, Honghai Liu, Honggen Du, Yixuan Sheng, Shao Chen

**Affiliations:** 1 The First Affiliated Hospital of Zhejiang Chinese Medical University (Zhejiang Provincial Hospital of Chinese Medicine), Hangzhou 310006, China; 20213030@zcmu.edu.cn (C.L.); 20233181@zcmu.edu.cn (K.W.); 20243109@zcmu.edu.cn (X.C.); 20193060@zcmu.edu.cn (Y.S.); 19963024@zcmu.edu.cn (H.D.); 2 State Key Laboratory of Robotics and System, School of Biomedical Engineering, Harbin Institute of Technology, Harbin 150001, China; 25b963012@stu.hit.edu.cn (B.M.); honghai.liu@hit.edu.cn (H.L.)

**Keywords:** multimodal physiological sensing, electroencephalography, high-density electromyography, cortico-muscular coupling, neurophysiological biomarker, muscle fatigue, adolescent idiopathic scoliosis

## Abstract

Adolescent idiopathic scoliosis (AIS) is associated with central neural control and peripheral muscle abnormalities. However, dynamic cortico-muscular coupling (CMC) during fatigue remains poorly understood. We used synchronous 32-channel electroencephalography and 32-channel high-density electromyography (HD-EMG) to record data from 20 adolescents with AIS and 20 healthy controls during the Biering–Sørensen test. Wavelet coherence and topographic mapping were used to characterize CMC and its spatial distribution. Descriptive analyses indicated group- and fatigue-related patterns in HD-EMG activation, cortical connectivity, and CMC topographies. For the prespecified C3/C4 regional analyses, effect sizes, 95% confidence intervals, exact *p* values, and false-discovery-rate-adjusted *p* values are reported to support transparent interpretation. These multimodal observations provide exploratory physiological patterns that require confirmation in larger studies with prespecified primary outcomes.

## 1. Introduction

Adolescent idiopathic scoliosis (AIS) is a complex, three-dimensional spinal deformity of unknown etiology that typically occurs around puberty, with an estimated prevalence of approximately 2–4%, thereby representing one of the most prevalent spinal disorders among adolescents [1,2]. Beyond the spinal curvature, AIS may lead to global postural imbalance, reduced spinal flexibility, and impaired biomechanical efficiency, with potential effects on cardiopulmonary function and psychosocial health, thereby potentially disrupting normal physical and psychological maturation in adolescents [3]. Currently, the clinical evaluation and follow-up of AIS rely primarily on radiographic parameters, such as the Cobb angle [2].

However, these static structural indices exhibit limited sensitivity for early detection and are suboptimal for accurately predicting disease progression [4]. Consequently, increasing attention has been directed toward identifying objective and quantifiable biomarkers with improved specificity and sensitivity through genetic, endocrine, and neuromuscular analyses [5].

Accumulating evidence suggests that AIS is not solely a structural skeletal disorder but rather a multifactorial condition involving dysfunction of both central neural regulation and peripheral muscular systems [6]. Non-invasive biosensors enable repeated, synchronized assessments of neural and muscular signals during movement tasks. Studies employing electroencephalography (EEG) have reported altered cortical oscillatory dynamics across multiple frequency bands in individuals with AIS [7]. Formaggio et al. identified elevated θ-band activity in the central cortical regions accompanied by asymmetric α-band lateralization within the sensorimotor areas in adolescents with AIS during postural tasks [8]. At the peripheral level, Wang et al. reported asymmetric back muscle activation and disrupted muscle synergies, assessed using high-density electromyography (HD-EMG) functioning as a high-resolution spatial sensor array, particularly on the concave side of the spinal curvature [9]. While these findings suggest concurrent alterations in central and peripheral neuromuscular function, most previous studies have examined neural and muscular changes in isolation, and the integrative functional relationship between cortical activity and muscle output remains poorly characterized.

Beyond EEG and EMG, AIS biosignal research encompasses radiographic and body-surface measures of spinal morphology, wearable or laboratory-based motion and postural control recordings, and muscle activation assessments. These modalities capture complementary structural, kinematic, and neuromuscular aspects of the condition [4,5,9]. However, they do not directly quantify the dynamic communication between cortical activity and peripheral muscle output during a standardized challenge. Recent EEG microstate research has likewise suggested altered temporal brain-state organization in AIS [10].

Cortico-muscular coupling (CMC) analysis provides an advanced quantitative framework for assessing functional connectivity between the central and peripheral neural systems [11]. By processing data derived from these dual-modal biosensors, CMC reflects the degree of synchronization between cortical oscillatory signals and the corresponding muscle activity across both the temporal and frequency domains. This serves as a neurophysiological indicator of functional synchronization along the corticospinal–muscular pathway and may reflect central–peripheral neural communication efficiency [12]. CMC has been widely applied in studies on motor control, neuromuscular disorders, and pain-related conditions [13]. However, its application in the context of AIS remains scarce and underexplored [14]. Further investigation of CMC using advanced sensing techniques in AIS may enable the identification of sensitive neurophysiological biomarkers and provide mechanistic insights into disease pathophysiology.

Muscle fatigue represents a common physiological stress condition encountered during daily postural maintenance and functional activities and can substantially influence sensory feedback, motor unit recruitment strategies, and central neural processing [15,16]. The Biering–Sørensen test (BST) is a well-established, standardized isometric endurance protocol that targets the trunk extensor musculature. Its isometric and quasi-static nature ensures high reproducibility and minimizes motion-related artifacts, thereby providing favorable signal acquisition conditions for simultaneous EEG and HD-EMG recordings [17,18]. Importantly, fatigue-related trunk muscle dysfunction appears to be clinically relevant in patients with AIS. Nguyen et al. reported differences in trunk muscle endurance between a group of female adolescents with AIS and a non-AIS matched healthy control (HC) group using the BST, thereby supporting the relevance of fatigue-based assessment in this population [19]. Given the baseline alterations in neural function reported in AIS, CMC responses captured by multi-channel sensors under fatigue conditions may exhibit distinct and potentially diagnostic characteristics. Therefore, the BST provides a methodologically appropriate paradigm for probing neural control mechanisms in AIS [20].

In this study, we used a synchronous dual-modal sensing system comprising a 32-channel EEG and a 32-channel HD-EMG array, together with continuous wavelet coherence analysis, to characterize cortico-muscular patterns during a fatigue-inducing BST paradigm. The objectives were to characterize complementary HD-EMG spatial maps, EEG connectivity maps, and CMC topographies; to compare AIS and HC patterns across early stable and fatigue phases; and to examine regional C3/C4 estimates with appropriate effect-size and multiple-comparison reporting. As an additional methodological check, channel-wise HD-EMG CMC analyses were conducted to assess whether the global grid aggregation obscured the material within-grid patterns.

## 2. Materials and Methods

### 2.1. Participants

Forty adolescents (20 with AIS and 20 HCs) were recruited for this study. Participants were prospectively enrolled at the First Affiliated Hospital of Zhejiang Chinese Medical University (Zhejiang Provincial Hospital of Chinese Medicine). The inclusion criteria for the AIS group were as follows: (1) radiographic confirmation of scoliosis with a Cobb angle > 10°; (2) age between 10 and 18 years; and (3) absence of concomitant neurological or musculoskeletal disorders that could affect motor function. HCs were recruited from local schools and communities and had no history of spinal deformities, neurological diseases, or chronic musculoskeletal disorders. The baseline characteristics of the participants are presented in Table 1. All participants and their legal guardians provided written informed consent before participating in the study. The study protocol was approved by the Ethics Committee of the First Affiliated Hospital of Zhejiang Chinese Medical University (approval number: 2023-K-303-01) and registered at ClinicalTrials.gov (NCT06758115).

The AIS and HC groups were numerically balanced (20 participants per group). This observational study did not involve a classification model; therefore, no class weighting, resampling, or other machine learning class balancing procedures were applicable.

### 2.2. HD-EMG and EEG Acquisition

For each participant, EEG and HD-EMG signals were synchronously acquired using a Twente Medical Systems International Reference Amplifier (TMSi REFA) system (TMS International B.V., Oldenzaal, The Netherlands). The saved analysis datasets had a sampling frequency of 2000 Hz.

**Figure 1:**
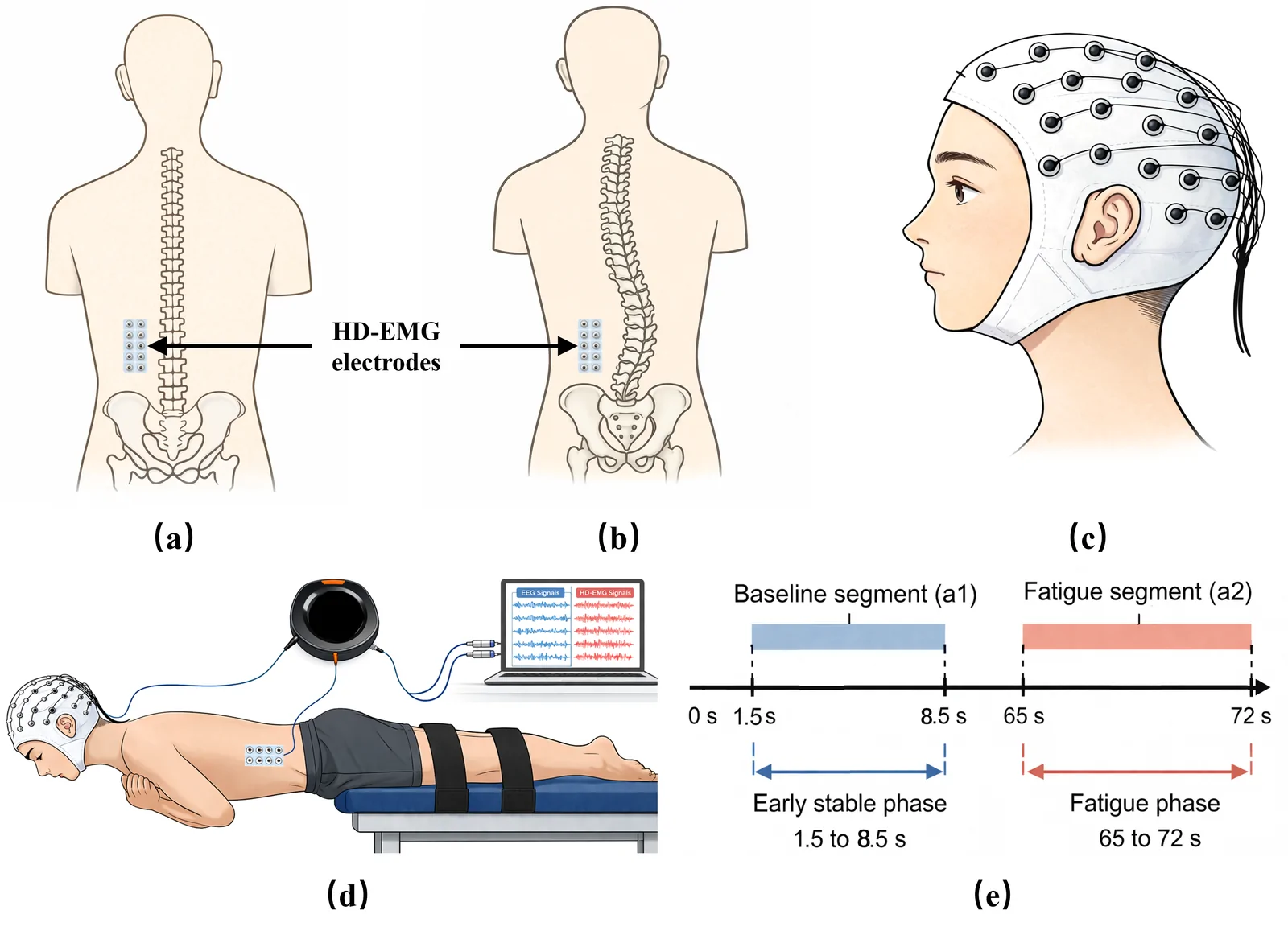
Experimental setup and temporal windows. (**a**) Placement of a 32-channel high-density electromyography (HD-EMG) electrode array over the paraspinal muscles in the healthy control group. (**b**) HD-EMG placement at an anatomically corresponding location on the concave side at the apex of the lumbar curvature in the adolescent idiopathic scoliosis group. (**c**) Electroencephalography (EEG) electrode configuration based on the international 10–10 system. (**d**) Synchronous EEG and HD-EMG recording during the Biering–Sørensen test. (**e**) Definition of the early stable (a1) and fatigue (a2) analysis windows.

HD-EMG signals from the paraspinal muscles were acquired using a 32-channel high-density surface biosensor array. In the HC group, these surface biosensors were placed 1.5 cm lateral to the spinous processes at the L1–L3 level on the left side (Figure 1a). In the AIS group, the sensor array was positioned at the corresponding location on the concave side at the apex of the lumbar curvature to capture curve-specific asymmetry in muscle activity (Figure 1b) [21]. EEG signals were recorded using 32 scalp sensor electrodes arranged according to the international 10–10 system, covering the bilateral frontal, central, parietal, and occipital cortices (Figure 1c).

The concave-side placement was selected a priori because AIS literature reports side-specific paraspinal muscle alterations, including concavity-related changes in muscle composition and activation [21]. This curve-specific sampling strategy was intended to characterize the local paraspinal region at the curve apex rather than to support a bilateral whole-trunk comparison. The exact 10–10 electrode positions were Fp1, Fpz, Fp2, F7, F3, Fz, F4, F8, FC5, FC1, FC2, FC6, M1, T7, C3, Cz, C4, T8, M2, CP5, CP1, CP2, CP6, P7, P3, Pz, P4, P8, POz, O1, Oz, and O2.

### 2.3. Experimental Task

All experiments were conducted at a controlled ambient temperature of approximately 24 °C. The participants were instructed to perform the BST in strict accordance with standardized procedures (Figure 1d) [22]. The participants were positioned prone on a bed approximately 1 m high, with the upper body extending beyond the bed edge. The anterior superior iliac spine was aligned with the edge of the support surface, whereas the lower body distal to the iliac crest was stabilized using fixation straps. The arms were crossed over the chest, with each hand placed on the opposite shoulder. Participants were required to maintain the trunk in a horizontal position parallel to the ground, sustaining the posture for 60–90 s or until task failure, defined as a trunk deviation of approximately 5–10° or greater from the horizontal plane. Prior to formal data acquisition, all participants underwent standardized familiarization trials to ensure correct posture and understanding of the tasks. Additionally, participants were instructed to avoid strenuous physical activity within 24 h prior to testing to minimize variability in fatigue response.

### 2.4. EEG and HD-EMG Analysis

All EEG and HD-EMG preprocessing and analyses were performed in MATLAB (R2023a, MathWorks, Natick, MA, USA), primarily using the EEGLAB toolbox for electrophysiological signal processing. EEG preprocessing used extended independent component analysis implemented in EEGLAB, with ICLabel-assisted component identification; the software and classifier are described in [23,24].

#### 2.4.1. Preprocessing

Following synchronization, the EEG and HD-EMG signals were independently preprocessed. EEG data used a common reference and were notch-filtered at 50 Hz (and harmonics), followed by 1–49 Hz band-pass filtering. Extended Infomax independent component analysis (runica, extended = 1) was applied in EEGLAB, and ICLabel-assisted component classification was used to identify artifact-related components; selected components were removed using the EEGLAB pop_subcomp procedure [23,24]. The cleaned EEG signals were subsequently normalized channel-wise to enhance inter-channel comparability.

HD-EMG signals were band-pass filtered between 20 and 150 Hz to attenuate low-frequency motion-related fluctuations and high-frequency noise. For the primary CMC analysis, the 32 filtered HD-EMG channels were spatially averaged to yield a representative global paraspinal muscle signal. This aggregation provided a robust grid-level measure for the coupling analysis but did not preserve local spatial heterogeneity; this issue was evaluated in the channel-wise validation reported in Supplementary Figures S1–S6.

The retained HD-EMG processing archive did not document an HD-EMG-specific 50 Hz notch operation. Therefore, we conducted a post hoc quality control and sensitivity assessment without altering the primary analysis. Each of the 32 HD-EMG channels in every retained 7 s epoch was screened for non-finite values and zero variance. A descriptive 50 Hz screen quantified power in the 49.5–50.5 Hz interval relative to the local 45–55 Hz spectral background, and channel epochs exceeding the cohort median plus six median absolute deviations were identified. Sensitivity CMC estimates were calculated after excluding flat/non-finite channels, after additionally excluding line-screened channel epochs, and after zero-phase removal of the fitted 50 Hz sinusoidal component from the quality-controlled global HD-EMG signal. No repeated trials were acquired; the a1 and a2 epochs represent distinct task states rather than replicates.

EEG and HD-EMG were recorded using distinct sensor arrays and processed using modality-specific filters and artifact-reduction procedures. These design choices were intended to minimize cross-modal contamination. Because dedicated leakage-resistant or surrogate data analyses were not available in the retained analysis archive, the magnitude of any residual influence could not be quantified and is acknowledged as a methodological limitation.

For spatial robustness validation, the CMC was calculated separately for each of the 32 processed HD-EMG channels using the same preprocessing and wavelet coherence procedure as in the primary analysis. Channel-wise CMC was summarized by its within-grid mean, median, and coefficient of variation; isolated invalid electrode values were omitted only from the corresponding summary.

To minimize the influence of fatigue accumulation and posture instability, CMC analysis focused on two predefined temporal segments: an early stable phase from 1.5 to 8.5 s after task onset (baseline segment, a1) and a later phase from 65 to 72 s (fatigue segment, a2). These segments were used for pre–post-fatigue comparisons (Figure 1e).

Each CMC epoch was 7 s long. The early window (a1, 1.5–8.5 s) excluded the initial task-onset adjustment period, whereas the later window (a2, 65–72 s) sampled the fatigue phase while remaining 18 s before the common 90 s task endpoint. All participants completed the 90 s recording, ensuring the availability of both windows. The fixed 7 s duration was selected to retain temporal specificity while providing a stable within-window estimate of CMC.

#### 2.4.2. CMC Analysis Based on Continuous Wavelet Transform

To quantify the time–frequency synchronization between cortical activity and muscle output, continuous wavelet transform combined with wavelet coherence (WCOH) analysis was applied to the EEG and HD-EMG signals. WCOH provides an effective signal-processing framework for analyzing multi-channel, nonstationary electrophysiological signals, enabling the simultaneous characterization of dynamic coupling across temporal and spectral domains, and is particularly suitable for analyzing complex electrophysiological signals.

The WCOH was estimated using a complex Morlet mother wavelet with a dimensionless central frequency of ω0 = 6. The frequency representation used for band averaging comprised 250 bins spanning 1–50 Hz, and the wavelet cross-spectrum was smoothed in time and scale before coherence normalization.

For each participant, the WCOH was calculated between individual EEG channels and the averaged HD-EMG signal, providing channel-specific estimates of cortico-muscular interaction. Specifically, let x(*t*) denote the EEG signal and y(*t*) denote the EMG signal. Both signals were transformed using analytic Morlet wavelets to obtain their time–frequency representations:

(1) $$\begin{array}{c} W_{x}(t,f)=\int_{-\infty }^{+\infty }x(\tau )\;\psi_{f}^{*}\left(\tau -t\right)\;d\tau \end{array}$$

The cross-wavelet spectrum between the EEG and EMG signals was then calculated as:

(2) $$\begin{array}{c} W_{xy}(t,f)=W_{x}(t,f\;)\;W_{y}^{*}(t,f) \end{array}$$

where ∗ denotes complex conjugation.

WCOH was defined as the normalized measure of the cross-wavelet spectrum relative to the auto-spectral power of each signal, given by:

(3) $$\begin{array}{c} {\mathrm{WCOH}}_{xy}(t,f)=\frac{{\left|S\left(W_{xy}(t,f)\right)\right|}^{2}}{S\left({\left|W_{x}(t,f)\right|}^{2}\right)\;S\left({\left|W_{y}(t,f)\right|}^{2}\right)} \end{array}$$

where *S*(·) represents a smoothing operator applied in both the time and frequency domains to enhance estimation robustness and reduce variance.

WCOH values range from 0 to 1, with higher values indicating stronger phase-locked synchronization between EEG and EMG signals at specific time–frequency coordinates.

#### 2.4.3. Frequency Band Segmentation and Feature Extraction

Based on the WCOH analysis, spatially resolved features were extracted within canonical neurophysiological frequency bands. Coherence values were averaged across frequency ranges corresponding to δ (1–4 Hz), θ (4–8 Hz), α (8–13 Hz), β (13–30 Hz), and low γ (30–45 Hz), enabling band-specific characterization of cortico-muscular interactions. These ranges follow conventional EEG frequency-band nomenclature [25].

At the muscular level, the preprocessed 32-channel HD-EMG signals were averaged across the defined analysis time window and reconstructed into two-dimensional spatial distribution maps based on the sensor array geometry, allowing the visualization of regional muscle recruitment patterns.

At the cortical level, functional connectivity was quantified by computing inter-channel EEG coherence within each frequency band, and connectivity matrices were constructed, where EEG channels served as network nodes and coherence values as edge weights, facilitating a graph-based representation of cortical network organization [26]. For anatomical interpretation, the EEG channels were grouped into functionally relevant cortical regions. The anterior regions included the frontopolar (Fp1, Fpz, and Fp2), frontal (F3, Fz, F4, F7, and F8), and fronto-central areas (FC5, FC1, FC2, and FC6). The central-related regions comprised the central (C3, Cz, and C4), temporal (T7 and T8), centro-parietal (CP5, CP1, CP2, and CP6), and parietal (P7, P3, Pz, P4, and P8) regions. The posterior regions included the parieto-occipital (POz) and occipital (O1, Oz, and O2) regions.

**Figure 2:**
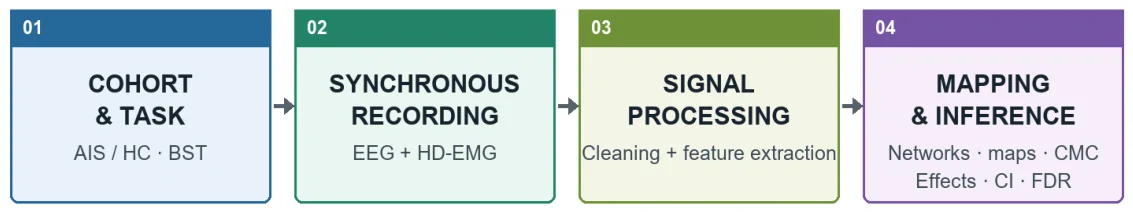
Study and analysis workflow. Participants with adolescent idiopathic scoliosis (AIS) and healthy controls (HC) completed the Biering–Sørensen test (BST) during synchronous electroencephalography (EEG) and high-density electromyography (HD-EMG). Signals were preprocessed, analyzed for HD-EMG spatial patterns, EEG connectivity, and cortico-muscular coupling (CMC), and summarized using regional C3/C4 estimates and channel-wise HD-EMG validation. CI, confidence interval; FDR, false discovery rate.

For the CMC analysis, the frequency-specific average coherence values between each EEG channel and the global HD-EMG signal were computed. These channel-level metrics were then projected onto scalp topographic maps, providing a spatial representation of the CMC strength. For channel selection, this study focused on C3 and C4 as the primary regions of interest. In recent EEG–EMG studies, C3 and C4 have commonly been regarded as approximations of the bilateral sensorimotor cortices, and central motor-related electrodes are frequently used to characterize CMC during motor and postural control tasks [27,28]. Furthermore, emerging evidence suggests that CMC over motor cortical regions is sensitive to both task demands and pathological conditions [29]. Given the growing body of evidence indicating altered neurophysiology and postural control mechanisms in AIS, focusing on C3 and C4 provides a pragmatic strategy to capture movement-related cortical drive pre- and post-fatigue and between groups while reducing redundancy and mitigating the multiple-comparison burden inherent in whole-scalp analyses. The study acquisition and analysis workflow is summarized in Figure 2.

### 2.5. Statistical Analysis

All statistical tests were two-sided with an alpha level of 0.05. For the prespecified C3 and C4 regional summaries, between-group comparisons at each time point used Mann–Whitney *U* tests, whereas within-group changes from a1 to a2 used Wilcoxon signed-rank tests. Exact *p* values, median differences, rank-biserial correlation effect sizes, and bootstrap 95% confidence intervals are reported. To account for the planned frequency-, region-, and condition-specific comparisons, the Benjamini–Hochberg false discovery rate (FDR) adjustment was applied separately to the 24 planned comparisons for each outcome family (CMC and brain network coupling strength). The unadjusted and FDR-adjusted results are presented in Supplementary Table S1. Whole-head topographies and connectivity maps were interpreted as descriptive group-level visualizations rather than electrode-wise or edge-wise inferential tests.

## 3. Results

### 3.1. Topographic Map Analysis of HD-EMG

**Figure 3:**
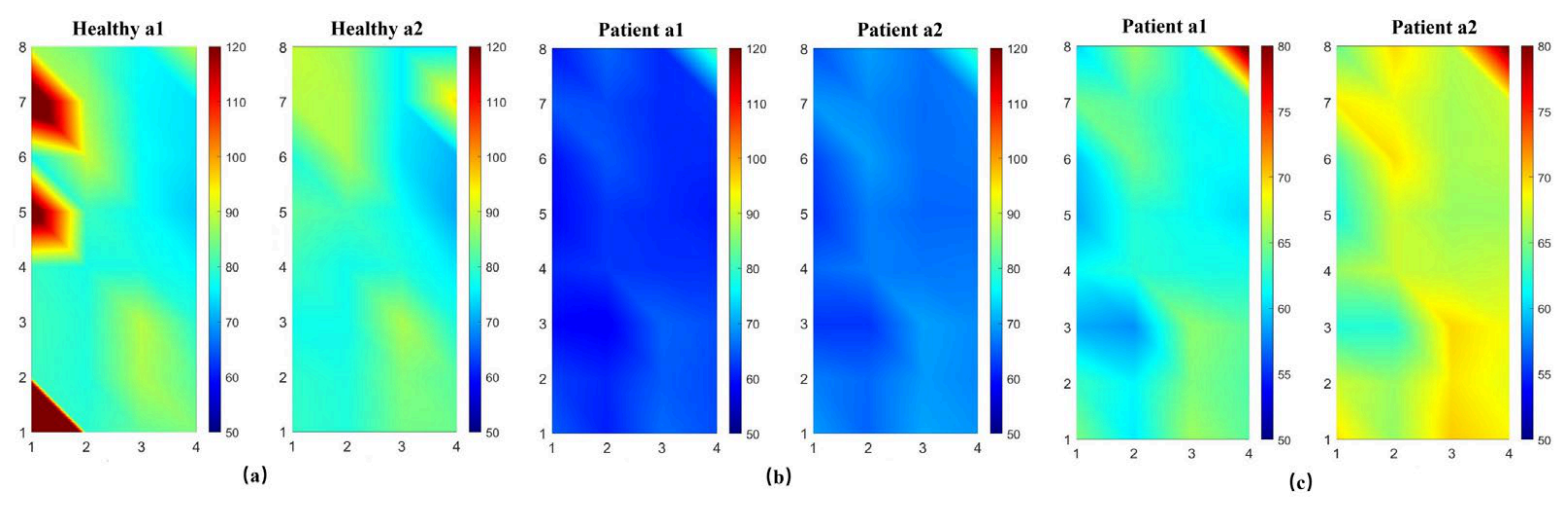
Group-level spatial activation patterns of high-density electromyography (HD-EMG) signals recorded from concave-side paraspinal muscles in healthy control and adolescent idiopathic scoliosis (AIS) groups before and after the Biering–Sørensen test (BST). (**a**,**b**) use a unified color scale to facilitate visual comparison between groups and phases; (**c**) uses an independent color scale to display the EMG spatial distribution. “Healthy” denotes the healthy control group and “Patient” denotes the AIS group. a1 denotes the early stable phase and a2 the fatigue phase. Warmer colors indicate higher EMG amplitude. These maps are descriptive group-level visualizations and do not represent electrode-wise inferential tests. The color bar in panel (**c**) explicitly represents this independent scale.

The spatial activation maps captured by the high-density surface sensor grid revealed distinct neuromuscular topographies, demonstrating that the spatial distribution of HD-EMG in the concave-side back muscles of adolescents with AIS differed from that of HC across both groups and fatigue conditions (Figure 3). During the BST fatigue task, the HC group exhibited relatively focal activation patterns with a well-defined spatial redistribution following fatigue. In contrast, the AIS group demonstrated reduced activation levels and impaired spatial organization at baseline; although the activation extent increased after fatigue, the overall distribution remained diffuse and poorly structured.

Before fatigue, back muscle EMG activity in the HC group was predominantly localized to a limited subset of electrode channels, forming a focal high-amplitude region with a clear spatial gradient, characterized by progressive attenuation toward adjacent channels. In contrast, the AIS group exhibited a relatively lower overall EMG activation amplitude than the HC group before fatigue, with an absence of high-activation clusters. The EMG amplitudes were more uniformly distributed across the channels than those in the HC group, with only mild local fluctuations and no obvious spatial gradient, indicating a more dispersed activation pattern in the concave-side paraspinal muscles in the AIS group.

Following BST-induced fatigue, the HC group exhibited a reduced overall EMG amplitude; however, the original high-amplitude activation regions expanded to adjacent channels, resulting in an increased activation area and reflecting a compensatory spatial redistribution pattern. In contrast, the AIS group showed an increased overall EMG amplitude post-fatigue, with more channels transitioning from low- to moderate- to high-amplitude levels. However, despite this increase in the extent of activation, the spatial pattern remained diffuse, with gradual inter-channel variation and persistent absence of a structured gradient, suggesting inefficient or uncoordinated muscle recruitment strategies.

### 3.2. Cortical Functional Connectivity Analysis

**Figure 4:**
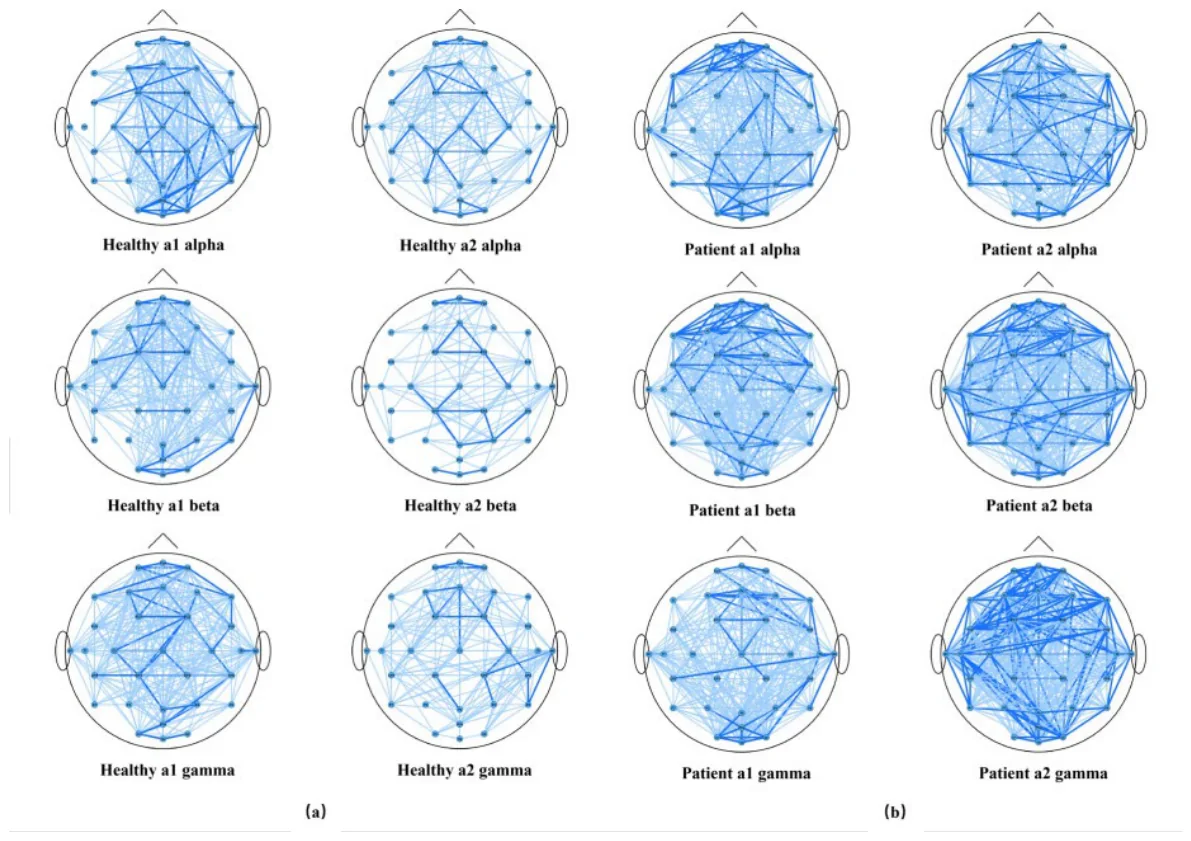
Descriptive whole-brain cortical functional connectivity networks derived from electroencephalography (EEG) coherence analysis in healthy controls and adolescent idiopathic scoliosis (AIS) groups before and after the Biering–Sørensen test. (**a**) Healthy control networks across the alpha, beta, and low gamma bands in the early stable (a1) and fatigue (a2) phases. (**b**) AIS networks across the same bands and phases. Edge thickness represents the coherence magnitude between the EEG channels; thicker edges indicate greater coherence. Electrodes are mapped to cortical regions according to the international 10–10 system. The networks visualize group-level patterns and are not interpreted as isolated edge-wise statistical tests.

The HC and AIS groups exhibited distinct cortical functional connectivity patterns across the *α*, *β*, and *γ* frequency bands pre- and post-fatigue (Figure 4). In both groups, whole-brain connectivity involving the anterior, central, and posterior regions was observed across the *α*, *β*, and *γ* bands pre- and post-fatigue; however, group differences were evident in both the predominant connectivity locations and the spatial extent of network distribution. The HC group demonstrated relatively localized and spatially constrained connectivity patterns both pre- and post-fatigue, whereas the AIS group exhibited a broader distribution of high-coherence connections at both time points and greater spatial dispersion post-fatigue than the HC group.

Before fatigue, in the *α* band, the HC group showed distributed connectivity across the anterior, central, and posterior regions, indicative of balanced network integration. In contrast, the AIS group demonstrated enhanced anterior–posterior connectivity with increased long-range interactions, particularly between the frontopolar, central, and posterior regions, suggesting augmented large-scale network synchronization. In the *β* band, the HC group exhibited predominantly localized connectivity between the anterior and central regions, consistent with task-relevant motor network engagement. Conversely, the AIS group showed more extensive anterior–central connectivity, with denser cross-regional interactions extending from the bilateral frontal to central regions than in the HC group, reflecting broader network recruitment. In the *γ* band, the HC group displayed limited anterior–posterior connectivity with a relatively restricted spatial extent. In contrast, the AIS group demonstrated the most pronounced long-range and interhemispheric connectivity, spanning the frontopolar, frontal, central, and posterior regions, indicative of heightened global network integration.

Following BST-induced fatigue, in the *α* band, the HC group demonstrated reduced connectivity compared to the pre-fatigue phase, with connections mainly retained in the central and posterior regions. In contrast, the AIS group maintained a relatively broad anterior–posterior connectivity pattern, with persistent connections between the frontopolar, central, and posterior regions. In the *β* band, the HC group exhibited the greatest degree of network contraction, with connectivity localized to the fronto-central, central, and centro-parietal regions. However, the AIS group did not show a comparable degree of contraction, and the connections between the anterior, central, and posterior regions remained relatively widespread. Although the HC group retained some anteroposterior connectivity in the γ band, the overall distribution was reduced. Conversely, the AIS group continued to exhibit the most extensive connectivity pattern among all frequency bands, with dense cross-regional connections persisting among the bilateral frontopolar, frontal, central, and posterior regions.

### 3.3. Cortico-Muscular WCOH Topography Analysis

**Figure 5:**
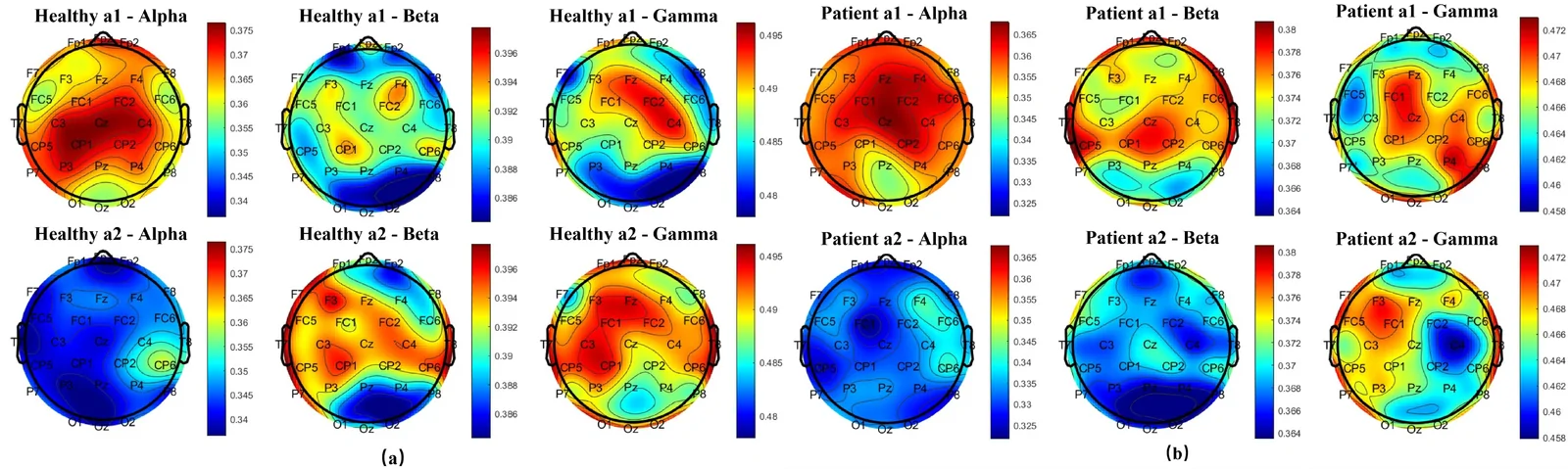
Descriptive scalp topographies of cortico-muscular coupling, quantified using wavelet coherence between electroencephalography and high-density electromyography, in healthy controls and adolescent idiopathic scoliosis (AIS) groups before and after the Biering–Sørensen test. (**a**) Healthy control group. (**b**) AIS group. a1 denotes the early stable phase and a2 the fatigue phase. Alpha, beta, and low gamma denote conventional neurophysiological frequency bands. The topographies visualize frequency-specific spatial characteristics of brain–muscle interaction; quantitative prespecified C3/C4 estimates and multiple-comparison-adjusted results are reported separately in Figure 5 and Supplementary Table S1.

The spatial distribution of cortico-muscular WCOH differed between the HC and AIS groups pre- and post-fatigue (Figure 5). During the BST fatigue task, both groups exhibited reductions in the *α*-band CMC, accompanied by changes in the *β*/*γ*-band coherence. However, the AIS group showed broader and more asymmetric spatial distributions across frequency bands, with more localized and lateralized coupling topographies, than those in the HC group.

Before fatigue, the CMC in the HC group within the α frequency band was primarily concentrated over the central and parietal channels, with higher coherence observed around the central–parietal regions (e.g., Cz, CP1/CP2, P3/P4). In contrast, *β*- and *γ*-band coupling showed relatively diffuse distributions or mild enhancement across the frontal–central regions at baseline. The AIS group demonstrated broader and more lateralized coupling distributions of pre-fatigue than the HC group. Additionally, the AIS group exhibited localized regions of elevated coherence across the *α*, *β*, and *γ* frequency bands, with spatial locations varying by frequency band and showing greater asymmetry than the HC group.

After fatigue induction, the HC group showed an overall reduction in *α*-band CMC, accompanied by the contraction of high-coherence regions. Concurrently, relative increases in *β*- and *γ*-band coherence emerged, primarily over the frontal and central channels (F/FC/C regions). The AIS group also exhibited reduced *α*-band coherence post-fatigue, whereas *β*- and *γ*-band coherence showed localized enhancement with more spatially concentrated distributions, particularly over the frontotemporal regions and the affected parietal area, with noticeable inter-channel asymmetry.

### 3.4. Changes in Central (C3/C4) Brain Network Coupling Strength and CMC Across Frequency Bands and Fatigue States

**Figure 6:**
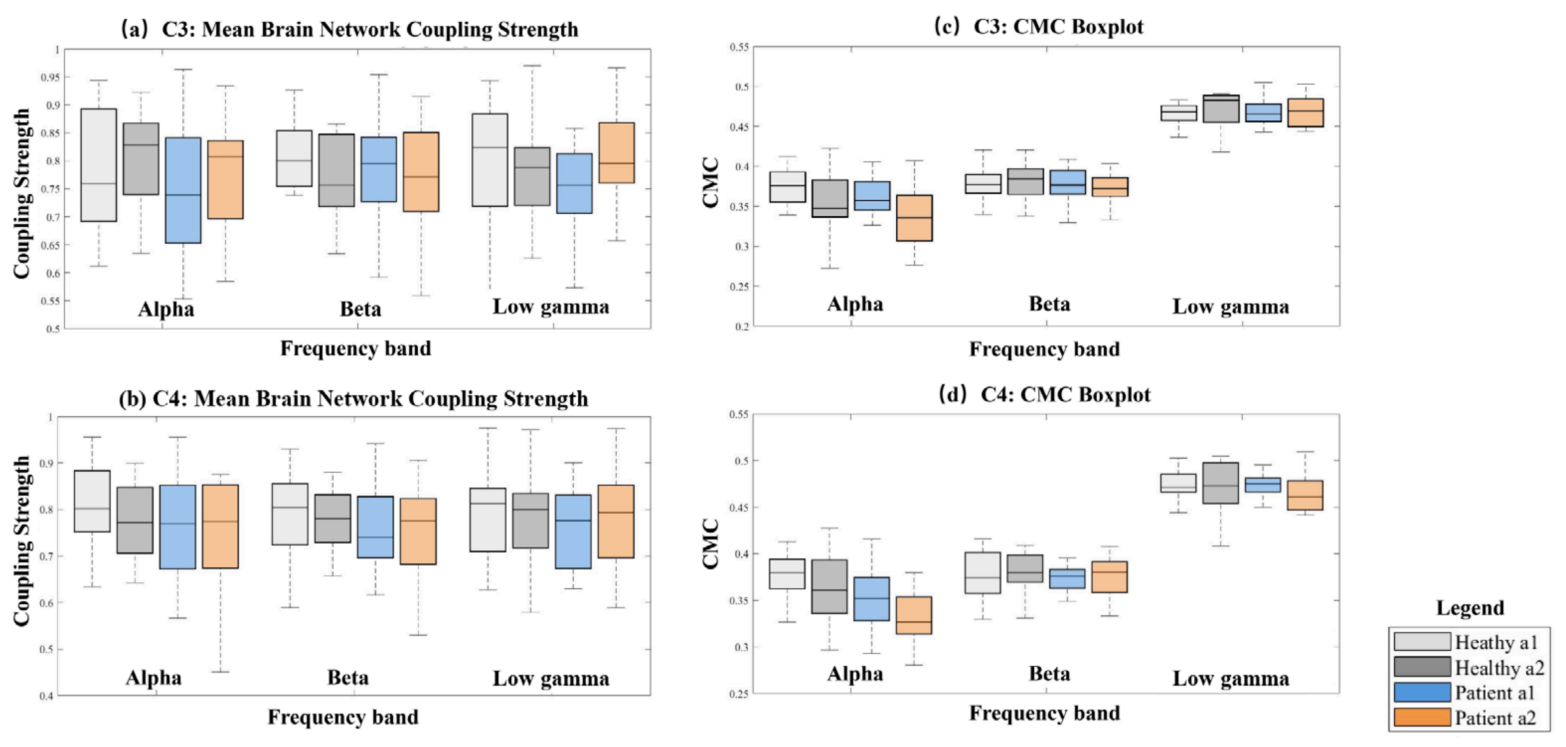
Descriptive distributions of average brain network coupling strength and cortico-muscular coupling (CMC) at C3 and C4 across frequency bands and fatigue states. (**a**) Mean brain network coupling strength at C3. (**b**) Mean brain network coupling strength at C4. (**c**) CMC at C3. (**d**) CMC at C4. a1 indicates the early stable phase and a2 the fatigue phase. “Healthy” represents the healthy control group and “Patient” the AIS group. Alpha, beta, and low gamma correspond to conventional neurophysiological frequency bands. Boxes represent the interquartile range, central lines the median, and whiskers the data range. Exact unadjusted and false discovery rate (FDR)-adjusted *p* values, effect sizes, and 95% confidence intervals are reported in Supplementary Table S1. No comparison remained statistically significant after FDR correction.

Figure 6 displays the descriptive distributions of the average brain network coupling strength and CMC at C3 and C4. These prespecified sensorimotor regions of interest were selected because they overlie cortical areas relevant to motor control and postural regulation [30]. The regional distributions were formally tested, whereas the whole-head maps were used only for descriptive spatial interpretation.

Descriptively, the brain network coupling strength varied across frequency bands and fatigue states. Some AIS distributions appeared lower than those of the HC group; however, the direction and magnitude of the differences varied by electrode, band, and phase. The C3 and C4 patterns were not identical, indicating mild descriptive lateral variation.

CMC distributions also showed frequency- and condition-dependent variations. Several unadjusted within-group comparisons suggested lower α-band CMC in the a2 phase. However, none of the prespecified C3/C4 comparisons remained statistically significant after FDR correction (all adjusted *p* ≥ 0.240 for CMC and all adjusted *p* ≥ 0.645 for brain network coupling strength; Supplementary Table S1). Therefore, the patterns in Figure 6 are interpreted as descriptive rather than confirmatory evidence of group or fatigue effects.

Channel-wise mean and median CMC summaries reproduced the principal relative patterns obtained with global HD-EMG aggregation. The global and channel-wise mean estimates showed high descriptive concordance (Spearman’s r = 0.992 at C3 and r = 0.993 at C4), and no stable focal within-grid CMC pattern was observed (Supplementary Figures S1–S6).

In the post hoc HD-EMG quality control audit, all 2560 channel epoch records were finite. Eighteen records (0.70%) had zero variance, and five (0.20%) exceeded the descriptive 50 Hz screen. Excluding flat channels yielded CMC estimates identical to those of the original global aggregation (Spearman’s r = 1.000). Excluding both flat and line-screened channel epochs remained highly concordant with the original estimates (r = 0.9998), as did zero-phase 50 Hz component removal after flat-channel exclusion (r = 0.9977). Across all sensitivity variants, none of the 24 prespecified C3/C4 comparisons remained significant after FDR correction (Supplementary Table S2).

## 4. Discussion

This study used a BST-induced static fatigue paradigm with synchronous EEG and HD-EMG recordings, WCOH analysis, and topographic mapping to provide an exploratory characterization of central–peripheral neuromuscular patterns in AIS. The integrated spatial, spectral, and regional observations provide a basis for hypotheses regarding fatigue-related brain–muscle interactions in AIS. Their clinical and mechanistic relevance should be evaluated in larger, prospectively designed studies with prespecified primary outcomes and balanced covariate structures.

### 4.1. Methodological Advantages of the BST Fatigue Paradigm for Cortico-Muscular Analysis

The BST was adopted to induce sustained isometric fatigue of the trunk extensor muscles, providing a standardized, reproducible, and controllable experimental condition for analyzing cortico-muscular interactions during fatigue [31]. From a physiological perspective, the BST specifically targets trunk extensor muscles that are frequently functionally compromised in AIS [32]. As isometric load accumulates, peripheral fatigue and associated afferent sensory feedback may induce adaptive neural responses at both the spinal and cortical levels, enabling the observation of the modulation of motor unit recruitment and muscle output [20]. From a methodological and engineering standpoint, the quasi-static nature of the BST minimizes motion-related artifacts, particularly those arising from head and trunk displacements. Crucially, this provides an optimal signal acquisition window for surface sensors, especially HD-EMG and EEG, which are highly susceptible to motion artifacts, thereby maximizing the signal-to-noise ratio of the acquired data and facilitating stable EEG acquisition. In addition, cortical oscillatory activity, particularly within the β and low γ frequency ranges, tends to remain relatively stable during sustained isometric contractions, which may enhance the reliability of CMC estimation [33]. The standardized posture, clearly defined termination criteria, and high reproducibility of the BST further ensured consistent task demands across participants, supporting between-group comparisons of neural control and fatigue regulation.

### 4.2. Spatial Characteristics of Paraspinal Muscle Activation Revealed by HD-EMG

HD-EMG findings showed that the HC group exhibited focal and spatially organized back muscle activation before fatigue, followed by a structured spatial redistribution of activation after fatigue. In contrast, the AIS group showed a lower activation amplitude and more diffuse spatial distribution of concave-side paraspinal muscle activity at baseline than the HC group, with no clear spatial reorganization post-fatigue. These observations indicate differences in muscle recruitment strategies during sustained isometric loading conditions between the AIS and HC groups. The spatial redistribution of muscle activation may reflect adaptive motor unit recruitment strategies during fatigue [34,35]. The absence of such redistribution in the AIS group may indicate reduced flexibility in peripheral neuromuscular regulation. These findings provide objective physiological references for the early identification of AIS-related neuromuscular alterations and may inform targeted rehabilitation approaches, including concave-side muscle strengthening and neuromuscular coordination training.

### 4.3. Cortical Functional Connectivity Reorganization Under Fatigue by EEG

Analysis of cortical functional connectivity revealed that the HC group maintained relatively concentrated connectivity patterns centered on the midline and sensorimotor-related regions, both pre- and post-fatigue. In contrast, the AIS group exhibited broader distributions of high-coherence connections at baseline, with further expansion and increased dispersion following fatigue. This pattern suggests greater recruitment of cortical regions in the AIS group to perform the same task, with fatigue associated with additional reorganization and expansion of functional connectivity networks. Previous EEG studies indicate that the spatial distribution of functional connectivity reflects task demands and neural resource allocation [36,37]. While increased coherence may indicate enhanced information transfer, excessive or spatially diffuse synchronization under fatigue conditions may also reflect reduced neural selectivity and regulatory efficiency [38,39]. This pattern may represent a compensatory neural strategy in individuals with AIS during postural control and motor tasks, supporting the maintenance of motor output stability. These connectivity characteristics may serve as potential neurophysiological markers for assessing motor control impairment and fatigue susceptibility in patients with AIS.

### 4.4. Altered Central–Peripheral Information Exchange Revealed by CMC

The descriptive CMC maps and regional distributions suggest frequency- and region-dependent reorganization of cortico-muscular interactions during the fatigue task. Possible contributors include altered postural control, asymmetric loading, and modified proprioceptive input in AIS [40]. However, these mechanisms remain hypotheses because the prespecified regional comparisons did not retain statistical significance after FDR correction. Related fatigue paradigms outside AIS have reported task-dependent changes in EEG–EMG network organization and CMC, supporting biological plausibility but not confirming the present descriptive patterns [41,42,43].

Although prior studies support fatigue-related changes in sensorimotor coupling [41,42,43], the present data do not provide confirmatory evidence of a specific α-band deficit or high-frequency compensatory mechanism in AIS. The directions of the descriptive changes differed across outcome type, electrode, and band. Larger studies with prespecified primary outcomes are required to test whether the observed patterns reflect altered central–peripheral communication or compensatory recruitment. Thus, within the local HD-EMG coverage and under the present quasi-static task, global HD-EMG aggregation preserved the principal CMC patterns, although this does not exclude more subtle spatial heterogeneity.

### 4.5. Strengths, Limitations, and Future Directions

Previous neurophysiological studies of AIS have largely focused on isolated EEG or EMG indicators, emphasizing either cortical rhythmic activity or muscle activation amplitude, while quantitative assessment of central–peripheral functional connectivity has remained limited. To the best of our knowledge, this study represents one of the earliest systematic investigations of CMC in AIS using synchronous EEG–HD-EMG acquisition under a BST-induced fatigue paradigm. By directly evaluating the functional coupling between cortical activity and muscle output, this study extends prior research toward integrated central–peripheral network analysis. In addition, HD-EMG combined with topographic mapping provides enhanced spatial resolution, enabling a more precise characterization of fatigue-related neuromuscular regulation [44]. The BST-based static fatigue paradigm further strengthens methodological rigor by minimizing motion artifacts and ensuring standardized loading conditions across participants.

In addition to mechanistic insights, brain–muscle coupling metrics may serve as candidate biomarkers for assessing disease severity and monitoring progression. Dynamic tracking of coupling indices may facilitate the evaluation of rehabilitation efficacy and optimization of training protocols. Furthermore, these findings may support neuromodulation-based interventions, including functional electrical stimulation, transcranial magnetic stimulation, or neurofeedback training, contributing to individualized rehabilitation strategies [45,46].

This study has some limitations. First, the sample size was relatively modest, and the cross-sectional design precluded causal inference regarding the relationship between the observed CMC alterations and AIS onset or progression. The sample size and multiple frequency-, electrode-, and condition-specific comparisons also limited the statistical power after FDR correction; therefore, visually apparent differences should not be interpreted as independently confirmed electrode-wise effects. Second, the analysis focused on concave-side paraspinal muscles without the inclusion of abdominal or contralateral muscle groups, which may limit the comprehensive assessment of trunk muscle coordination. Third, neural function assessment relied primarily on electrophysiological measures without integration of structural or functional neuroimaging, and the potential influences of genetic or endocrine factors were not explored.

The protocol used a single 32-channel array to prioritize local coverage of the predefined concave-side curve-apex paraspinal region; a simultaneous contralateral array was not used. Consequently, the present HD-EMG and CMC findings cannot establish bilateral muscle asymmetry or whole-trunk intermuscular coordination.

In addition, no dedicated leakage-resistant connectivity metric or surrogate-based EMG-contamination test was applied. Thus, despite synchronous acquisition with distinct sensor arrays, common reference EEG, modality-specific filtering, and independent component analysis-based artifact removal, residual EEG–EMG contamination or volume conduction effects cannot be completely excluded. Recording impedance values were not available in the retained analysis archive; future studies should prospectively document impedance thresholds and reference procedures.

The sex distribution was uneven (female: 17/20 [85.0%] in the AIS group and 12/20 [60.0%] in the HC group; Fisher’s exact *p* = 0.155). The female predominance in the AIS group is consistent with established epidemiological patterns, particularly for clinically larger curves [47]. Nevertheless, the unequal sex distribution between the present groups remains a potential confounder. The small number of male participants precluded reliable sex-stratified or covariate-adjusted analyses. With 20 participants per group, the study was sensitive primarily to large independent-group effects (approximately Cohen’s d ≥ 0.89 at 80% power, two-sided α = 0.05); smaller effects may not have been detected. Finally, CMC was estimated using predefined 7 s windows; alternative window lengths were not tested in the present study and should be examined in future sensitivity analyses.

Future studies should incorporate larger sample sizes and longitudinal follow-up designs to examine the dynamic evolution of CMC during disease progression and rehabilitation. Additionally, integrating multi-muscle recordings with multimodal imaging and kinematic analysis, as well as genetic and endocrine data, may enable the incorporation of molecular-level evidence and facilitate the construction of a multi-scale framework spanning molecular- to system-level mechanisms. These approaches may further elucidate the mechanisms underlying central–peripheral communication, providing stronger support for functional assessment and individualized interventions in patients with AIS. Although the current study utilized a laboratory-grade high-density sensing system, the ultimate goal of this sensing paradigm is clinical translation. If future studies confirm the exploratory spatial and spectral patterns reported in this study, their clinical relevance could be assessed using reduced-channel wearable sensing configurations. For example, future research could explore the integration of targeted biosensor configurations into wearable monitoring devices to support home-based tracking of neuromuscular function in AIS, although substantial engineering development and clinical validation would be required before such systems could be implemented.

Beyond AIS, recent EEG-based driver fatigue literature also illustrates the broader feasibility of using non-invasive EEG to characterize fatigue-related neural state changes; this contextual evidence should not be interpreted as disease-specific validation for AIS [48].

## 5. Conclusions

In 20 adolescents with AIS and 20 HCs, synchronous EEG–HD-EMG recording and CMC analysis provided a feasible framework for characterizing fatigue-related central–peripheral patterns. Post hoc HD-EMG quality control sensitivity analyses preserved the original CMC pattern. However, none of the prespecified C3/C4 comparisons remained statistically significant after FDR correction (CMC-adjusted *p* ≥ 0.240; brain network coupling-adjusted *p* ≥ 0.645). Therefore, the observed visual and regional trends require confirmation in larger studies with prespecified primary outcomes and appropriate multiplicity control before they can be considered candidate clinical indicators.

## Figures and Tables

**Table 1:** Baseline characteristics of the AIS and HC groups.

Baseline	AIS Group	HC Group	*p* Value
Age, mean (SD), years	15.30 ± 2.60	13.95 ± 1.99	0.073
Sex, no. (%)			
Female	17 (85.0)	12 (60.0)	0.155
Male	3 (15.0)	8 (40.0)	
Height, mean (SD), cm	164.75 ± 5.21	161.90 ± 13.75	0.391
Weight, mean (SD), kg	51.65 ± 11.26	51.55 ± 14.61	0.981
BMI, mean (SD), kg/m^2^	18.93 ± 3.35	19.26 ± 2.99	0.746
Cobb angle, mean (SD), ^°^	22.00 ± 5.83	-	-
ATR, mean (SD), ^°^	6.66 ± 2.88	-	-

BMI: body mass index (calculated as weight in kilograms divided by height in meters squared); ATR: trunk rotation angle; AIS group: adolescent idiopathic scoliosis group; HC group: healthy control group; SD: standard deviation.

## Data Availability

The original contributions presented in this study are included in the article/Supplementary Materials. Further inquiries can be directed to the corresponding authors.

## Supplementary Materials

The following supporting information can be downloaded at: https://www.mdpi.com/article/10.3390/bios16080451/s1. Supplementary Table S1: C3/C4 descriptive and inferential statistics, including exact *p* values, FDR-adjusted *p* values, rank-biserial effect sizes, and bootstrap 95% confidence intervals; Supplementary Table S2: HD-EMG quality control and CMC sensitivity-analysis summary, including channel epoch screening results, concordance with the original aggregation, and FDR-adjusted sensitivity comparisons; Figure S1: Comparison of global and channel-wise HD-EMG CMC estimates; Figure S2: Robust channel-wise median CMC estimates; Figure S3: Within-grid variability of channel-wise HD-EMG CMC; Figure S4: Concordance between global and channel-wise mean HD-EMG CMC; Figure S5: Spatial distribution of channel-wise CMC for C3; Figure S6: Spatial distribution of channel-wise CMC for C4.

## Abbreviations

The following abbreviations are used in this manuscript:

AIS	Adolescent idiopathic scoliosis
ATR	Trunk rotation angle
BMI	Body mass index
BST	Biering–Sørensen Test
CMC	Cortico-muscular coupling
EEG	Electroencephalography
HC	Healthy control
HD-EMG	High-density electromyography
IQR	Interquartile range
SD	Standard deviation
WCOH	Wavelet coherence
