# Supplementary material for: A Dual-Modal Biosensing Approach to Evaluate Cortico-Muscular Coupling in Adolescent Idiopathic Scoliosis
Source: Biosensors (Basel). 2026 Aug 20;16(8):451. doi: 10.3390/bios16080451 (PMC13511288; doi:10.3390/bios16080451)
Supplement: Supplementary file 1 [file biosensors-16-00451-s001.zip › biosensors-4366751-Supplementary S1.pdf]

Supplementary Materials

Channel-Wise HD-EMG CMC Validation and C3/C4 Statistical Results

Supplementary Statistical Methods. For the prespecified C3/C4 regional summaries, between-group comparisons used Mann–Whitney U tests and within-group a1-to-a2 changes used Wilcoxon signed-rank tests. Median differences, rank-biserial effect sizes, bootstrap 95% confidence intervals, exact p values, and Benjamini–Hochberg FDR-adjusted p values are reported. FDR correction was applied separately to the 24 planned comparisons in each outcome family. Whole-head maps were interpreted descriptively, without electrode-wise hypothesis tests. No comparison remained significant after FDR correction.

Supplementary Table S1. C3/C4 inferential statistics. The median difference is group 1 minus group 2 for between-group contrasts and a2 minus a1 for paired contrasts. Positive rank-biserial r indicates higher values in group 1 or at a2, respectively.

Outcome	Electrode	Band	Contrast	Test	Median difference	Raw p	FDR p	Rank-biserial r	95% CI
CMC	C3	alpha	HC vs AIS a1	Mann–Whitney U	0.0185	0.1988	0.5965	0.240	[-0.0099, 0.0333]
CMC	C3	alpha	HC vs AIS a2	Mann–Whitney U	0.0085	0.3507	0.7014	0.175	[-0.0134, 0.0522]
CMC	C3	alpha	HC a2 minus a1	Wilcoxon SR	-0.0217	0.0176	0.2403	-0.621	[-0.0488, -0.0004]
CMC	C3	alpha	AIS a2 minus a1	Wilcoxon SR	-0.0291	0.0400	0.2403	-0.524	[-0.0370, -0.0012]
CMC	C3	beta	HC vs AIS a1	Mann–Whitney U	-0.0023	0.7150	0.8421	-0.070	[-0.0224, 0.0160]
CMC	C3	beta	HC vs AIS a2	Mann–Whitney U	0.0117	0.3235	0.7014	0.185	[-0.0099, 0.0251]
CMC	C3	beta	HC a2 minus a1	Wilcoxon SR	-0.0028	0.6580	0.8312	0.116	[-0.0091, 0.0197]
CMC	C3	beta	AIS a2 minus a1	Wilcoxon SR	-0.0040	0.2790	0.6695	-0.276	[-0.0152, 0.0057]
CMC	C3	low gamma	HC vs AIS a1	Mann–Whitney U	0.0018	0.9246	0.9405	-0.020	[-0.0127, 0.0121]
CMC	C3	low gamma	HC vs AIS a2	Mann–Whitney U	0.0125	0.5609	0.8012	0.110	[-0.0174, 0.0258]
CMC	C3	low gamma	HC a2 minus a1	Wilcoxon SR	0.0091	0.2772	0.6695	0.284	[-0.0040, 0.0169]
CMC	C3	low gamma	AIS a2 minus a1	Wilcoxon SR	0.0002	0.7369	0.8421	0.086	[-0.0103, 0.0138]
CMC	C4	alpha	HC vs AIS a1	Mann–Whitney U	0.0265	0.0720	0.2882	0.335	[-0.0037, 0.0491]
CMC	C4	alpha	HC vs AIS a2	Mann–Whitney U	0.0257	0.0639	0.2882	0.345	[-0.0080, 0.0670]
CMC	C4	alpha	HC a2 minus a1	Wilcoxon SR	-0.0110	0.0910	0.3120	-0.442	[-0.0591, 0.0126]
CMC	C4	alpha	AIS a2 minus a1	Wilcoxon SR	-0.0293	0.0206	0.2403	-0.590	[-0.0467, -0.0087]
CMC	C4	beta	HC vs AIS a1	Mann–Whitney U	-0.0019	0.7972	0.8697	-0.050	[-0.0169, 0.0189]
CMC	C4	beta	HC vs AIS a2	Mann–Whitney U	-0.0012	0.4903	0.8012	0.130	[-0.0166, 0.0254]
CMC	C4	beta	HC a2 minus a1	Wilcoxon SR	0.0042	0.6009	0.8012	0.137	[-0.0071, 0.0101]
CMC	C4	beta	AIS a2 minus a1	Wilcoxon SR	0.0009	0.9405	0.9405	0.019	[-0.0096, 0.0115]
CMC	C4	low gamma	HC vs AIS a1	Mann–Whitney U	-0.0045	0.5979	0.8012	-0.100	[-0.0113, 0.0058]
CMC	C4	low gamma	HC vs AIS a2	Mann–Whitney U	0.0083	0.4903	0.8012	0.130	[-0.0120, 0.0271]
CMC	C4	low gamma	HC a2 minus a1	Wilcoxon SR	-0.0019	0.5461	0.8012	-0.158	[-0.0150, 0.0072]
CMC	C4	low gamma	AIS a2 minus a1	Wilcoxon SR	-0.0103	0.0366	0.2403	-0.533	[-0.0168, 0.0013]
Brain network	C3	alpha	HC vs AIS a1	Mann–Whitney U	0.0104	0.5075	0.8248	0.125	[-0.1017, 0.1705]
Brain network	C3	alpha	HC vs AIS a2	Mann–Whitney U	0.0263	0.4570	0.8248	0.140	[-0.0529, 0.1189]
Brain network	C3	alpha	HC a2 minus a1	Wilcoxon SR	-0.0010	0.6292	0.8248	0.126	[-0.0216, 0.0347]
Brain network	C3	alpha	AIS a2 minus a1	Wilcoxon SR	-0.0026	0.6009	0.8248	0.137	[-0.0469, 0.1255]
Brain network	C3	beta	HC vs AIS a1	Mann–Whitney U	0.0050	0.5792	0.8248	0.105	[-0.0428, 0.0844]
Brain network	C3	beta	HC vs AIS a2	Mann–Whitney U	-0.0096	0.7764	0.8470	-0.055	[-0.0993, 0.0821]
Brain network	C3	beta	HC a2 minus a1	Wilcoxon SR	-0.0060	0.1978	0.8248	-0.337	[-0.0652, 0.0012]
Brain network	C3	beta	AIS a2 minus a1	Wilcoxon SR	-0.0137	0.6813	0.8248	-0.105	[-0.0937, 0.0696]
Brain network	C3	low gamma	HC vs AIS a1	Mann–Whitney U	0.0629	0.1136	0.8248	0.295	[-0.0289, 0.1297]
Brain network	C3	low gamma	HC vs AIS a2	Mann–Whitney U	-0.0193	0.4407	0.8248	-0.145	[-0.1136, 0.0401]
Brain network	C3	low gamma	HC a2 minus a1	Wilcoxon SR	-0.0201	0.3547	0.8248	-0.242	[-0.0853, 0.0032]
Brain network	C3	low gamma	AIS a2 minus a1	Wilcoxon SR	0.0513	0.0269	0.6450	0.581	[0.0178, 0.0927]
Brain network	C4	alpha	HC vs AIS a1	Mann–Whitney U	0.0609	0.1636	0.8248	0.260	[-0.0312, 0.1690]
Brain network	C4	alpha	HC vs AIS a2	Mann–Whitney U	-0.0070	0.7557	0.8470	0.060	[-0.0967, 0.0997]
Brain network	C4	alpha	HC a2 minus a1	Wilcoxon SR	-0.0257	0.0910	0.8248	-0.442	[-0.0927, 0.0029]
Brain network	C4	alpha	AIS a2 minus a1	Wilcoxon SR	0.0087	1.0000	1.0000	0.000	[-0.0649, 0.0821]
Brain network	C4	beta	HC vs AIS a1	Mann–Whitney U	0.0491	0.6554	0.8248	0.085	[-0.0641, 0.1335]
Brain network	C4	beta	HC vs AIS a2	Mann–Whitney U	0.0164	0.5075	0.8248	0.125	[-0.0697, 0.1047]
Brain network	C4	beta	HC a2 minus a1	Wilcoxon SR	-0.0197	0.5461	0.8248	-0.158	[-0.0962, 0.0115]
Brain network	C4	beta	AIS a2 minus a1	Wilcoxon SR	-0.0031	0.6874	0.8248	-0.105	[-0.0562, 0.0325]
Brain network	C4	low gamma	HC vs AIS a1	Mann–Whitney U	0.0370	0.2616	0.8248	0.210	[-0.0839, 0.1363]

Outcome	Electrode	Band	Contrast	Test	Median difference	Raw p	FDR p	Rank-biserial r	95% CI
Brain network	C4	low gamma	HC vs AIS a2	Mann–Whitney U	0.0034	0.8604	0.8978	0.035	[-0.0712, 0.1001]
Brain network	C4	low gamma	HC a2 minus a1	Wilcoxon SR	-0.0097	0.4939	0.8248	-0.179	[-0.1028, 0.0019]
Brain network	C4	low gamma	AIS a2 minus a1	Wilcoxon SR	0.0326	0.2772	0.8248	0.286	[-0.0171, 0.0509]

Supplementary Methods. To assess whether averaging the 32 high-density electromyography (HD-EMG) channels before CMC estimation obscured a material spatial effect, CMC was additionally computed separately for each HD-EMG channel. The analysis used the same preprocessing, time windows, C3/C4 EEG electrodes, frequency bands, and CMC procedure as the primary analysis in 20 participants with AIS and 20 healthy controls. For each participant-condition combination, channel-wise CMC was summarized by its mean, median, and coefficient of variation across valid channels. Channels yielding isolated non-finite CMC values were excluded only from the corresponding spatial summary; no participant was excluded on this basis.

Abbreviations: AIS, adolescent idiopathic scoliosis; CMC, cortico-muscular coupling; FDR, false discovery rate; HC, healthy control; HD-EMG, high-density electromyography.

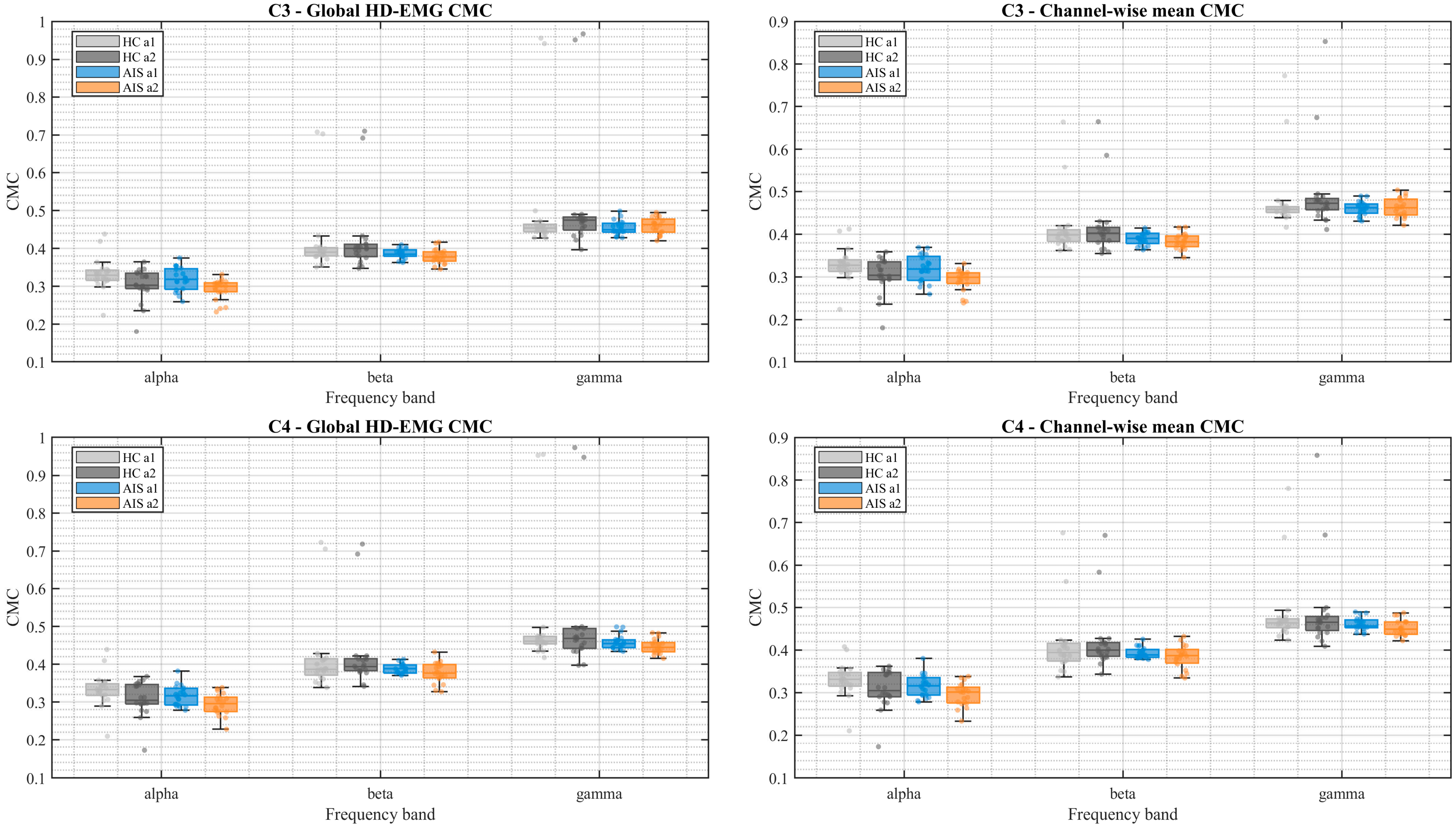

Figure S1. Comparison of global and channel-wise HD-EMG CMC estimates. CMC was calculated using the original global HD-EMG signal (left panels) or separately for each of the 32 HD-EMG channels followed by spatial averaging (right panels). Results are shown for C3 and C4 across alpha, beta, and low-gamma bands. Boxes show the interquartile range and the center line the median; dots denote individual participants.

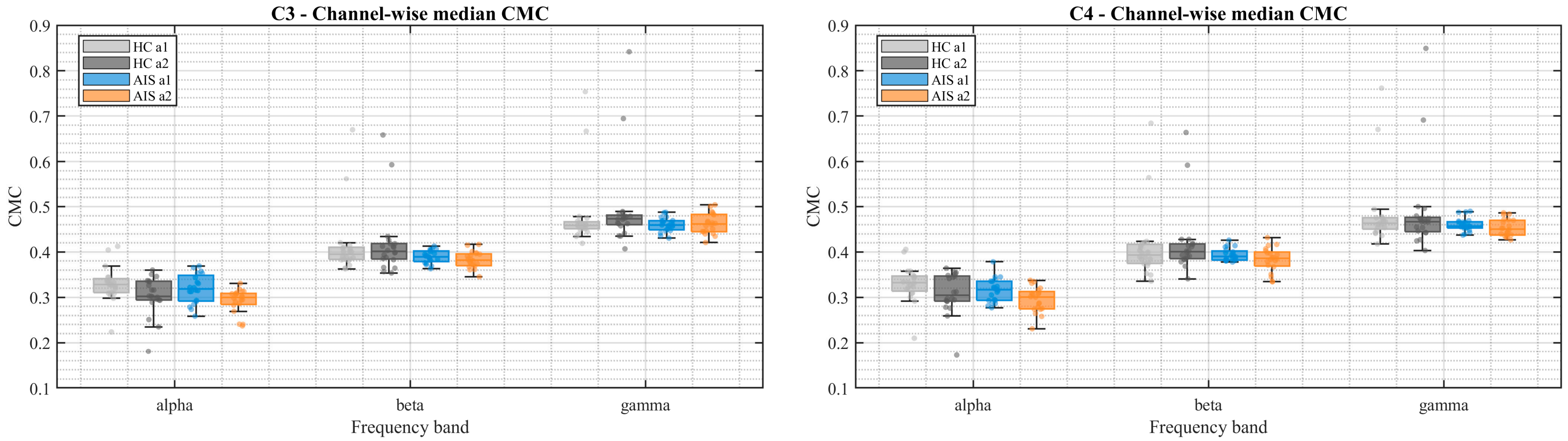

Figure S2. Robust channel-wise median CMC estimates. For each participant and condition, CMC was computed at each HD-EMG channel and summarized by the within-grid median. This analysis evaluates whether the findings are unduly influenced by a small number of channels. Boxes show the interquartile range and the center line the median; dots denote individual participants.

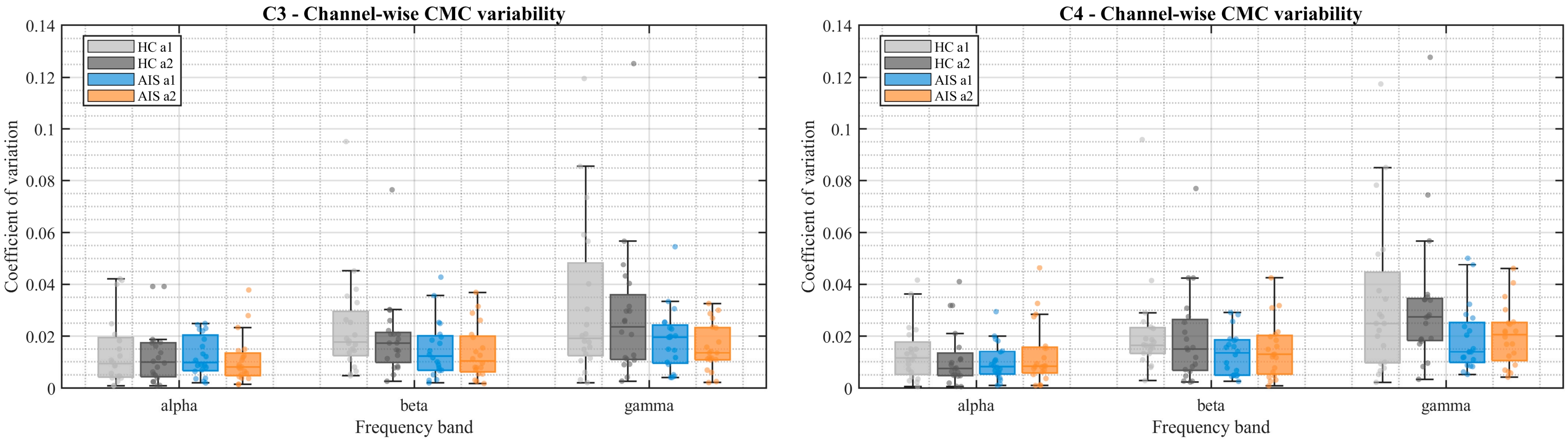

Figure S3. Within-grid variability of channel-wise HD-EMG CMC. The coefficient of variation was calculated across valid HD-EMG channels for each participant, time window, EEG electrode, and frequency band. Lower values indicate greater homogeneity of CMC within the local HD-EMG grid.

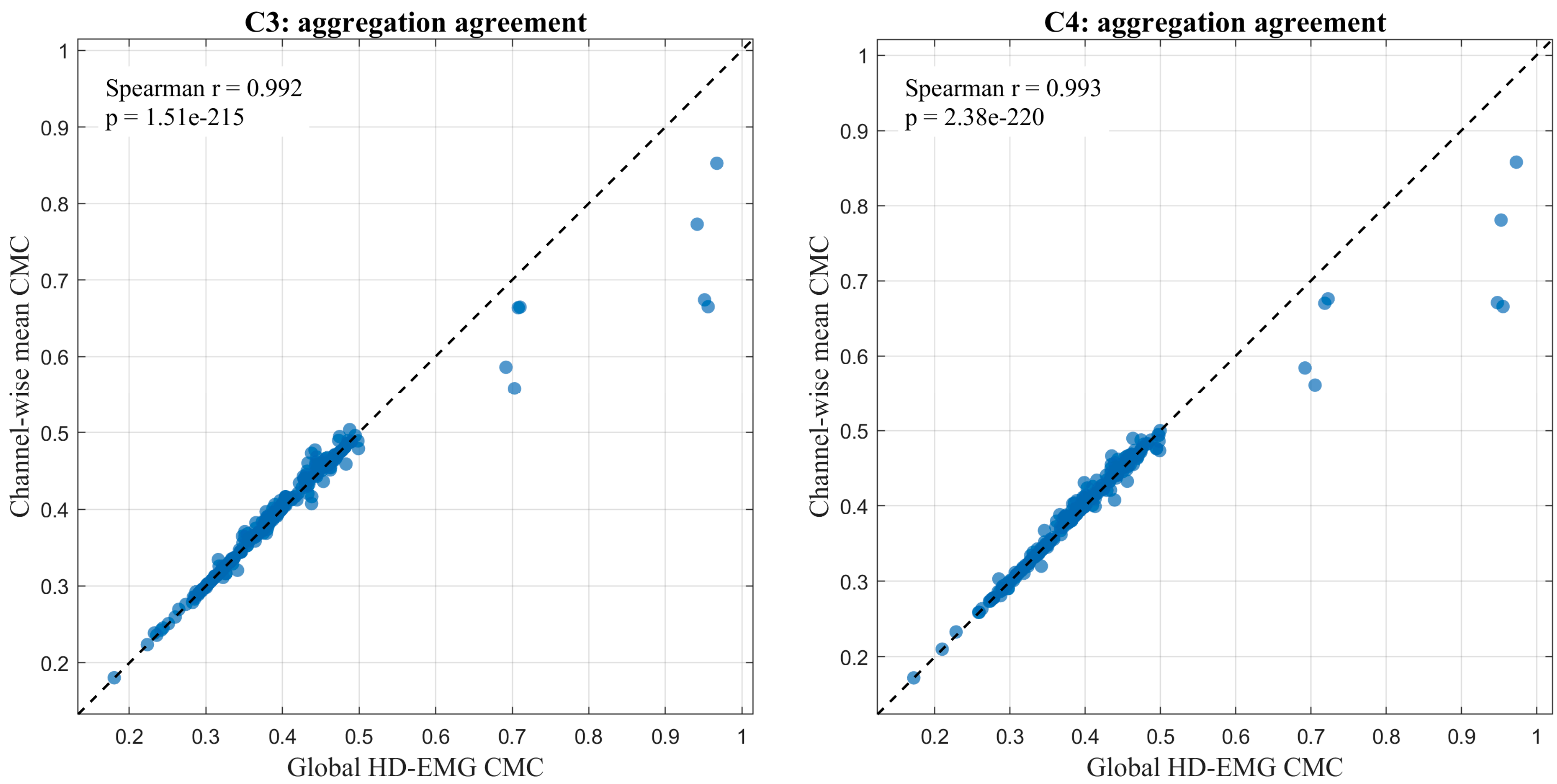

Figure S4. Concordance between global and channel-wise mean HD-EMG CMC. Each point represents one participant × time window × frequency-band observation. The dashed line indicates identity. Spearman correlations are descriptive measures of relative-pattern preservation between aggregation strategies.

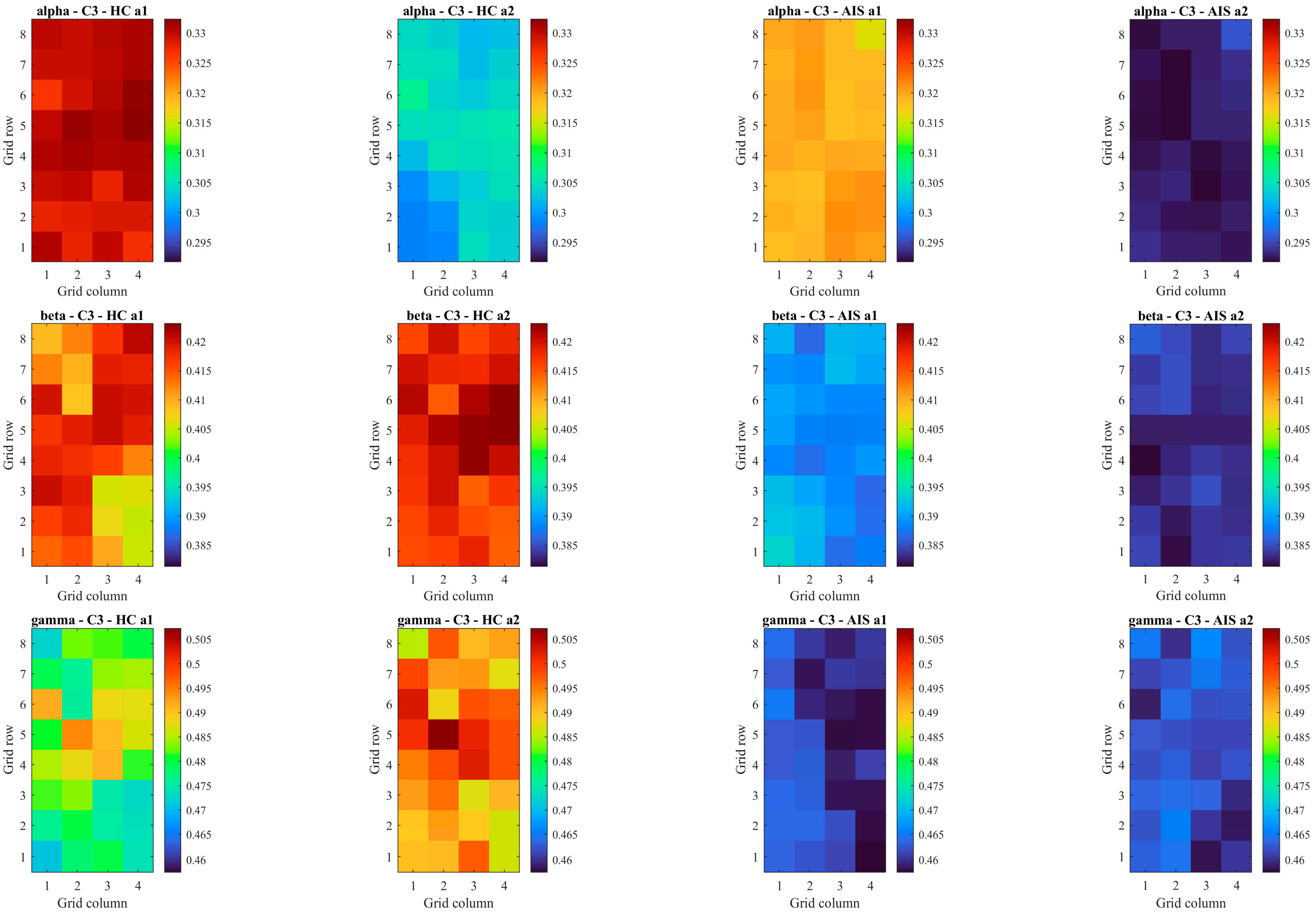

Figure S5. Spatial distribution of channel-wise CMC for the C3 EEG electrode. Each 8 × 4 map shows group-mean channel-wise CMC across the HD-EMG grid for one frequency band and condition. Grid rows and columns indicate relative array positions, not anatomical coordinates. Isolated invalid electrode values were excluded only from their corresponding channel-wise summaries.

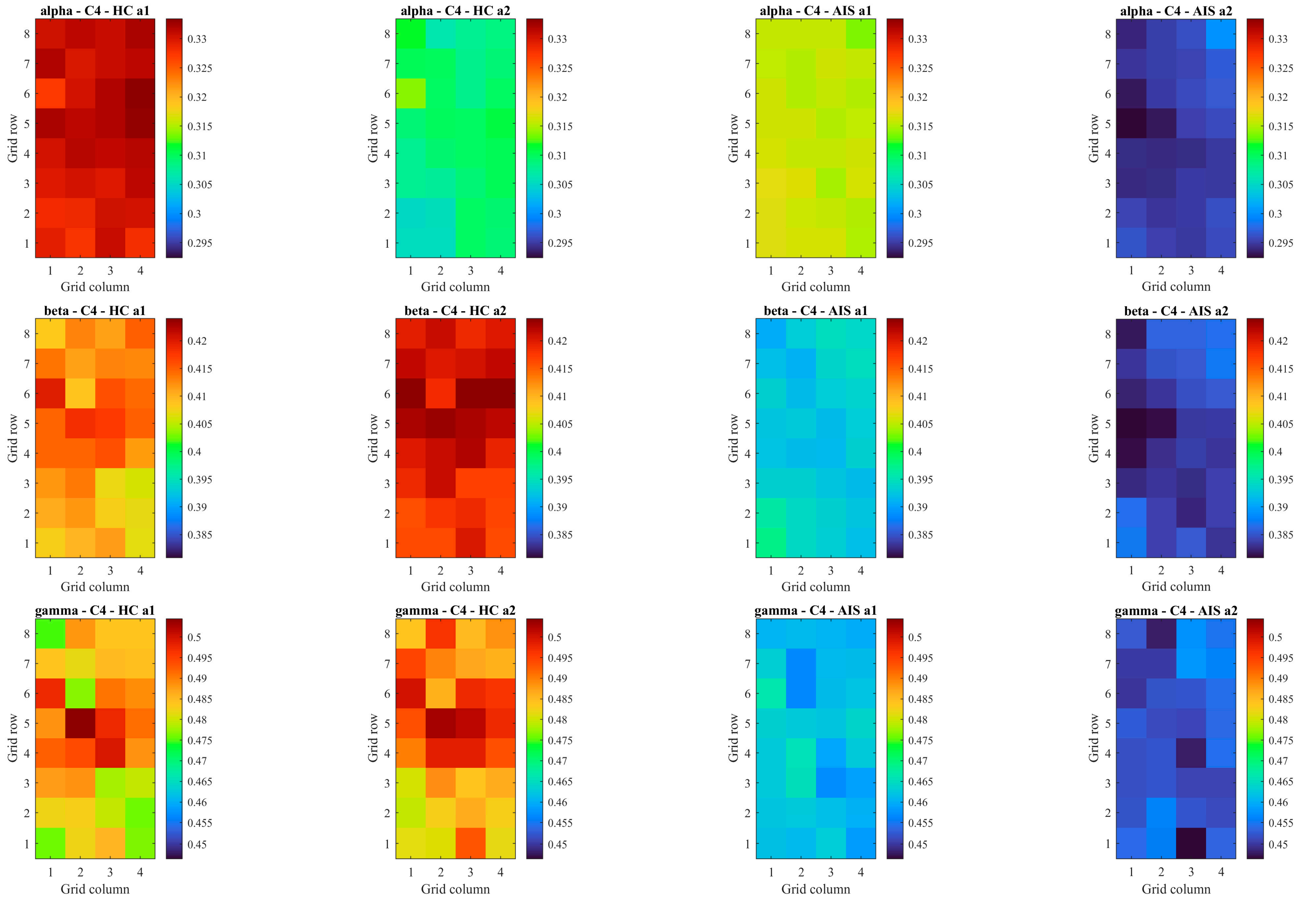

Figure S6. Spatial distribution of channel-wise CMC for the C4 EEG electrode. Each 8 × 4 map shows group-mean channel-wise CMC across the HD-EMG grid for one frequency band and condition. Grid rows and columns indicate relative array positions, not anatomical coordinates. Isolated invalid electrode values were excluded only from their corresponding channel-wise summaries.
