# Supplementary material for: A Dual-Modal Biosensing Approach to Evaluate Cortico-Muscular Coupling in Adolescent Idiopathic Scoliosis
Source: Biosensors (Basel). 2026 Aug 20;16(8):451. doi: 10.3390/bios16080451 (PMC13511288; doi:10.3390/bios16080451)
Supplement: Supplementary file 1 [file biosensors-16-00451-s001.zip › biosensors-4366751-Supplementary S2.pdf]

# Supplementary Materials for the Second-Round Revision

## HD-EMG Quality Control and CMC Sensitivity Analysis

This addendum reports only the additional analyses undertaken in response to the second-round reviewer comments. The supplementary materials submitted with the first-round revision remain unchanged.

**Methods for the post hoc HD-EMG quality control audit.**

The audit included 80 retained 7-s epochs from 40 participants across two task phases, yielding 2,560 HD-EMG channel epoch records. Records were screened for non-finite values, zero variance, and a descriptive 50-Hz spectral outlier criterion based on the 49.5–50.5 Hz to 45–55 Hz background power ratio (median plus six median absolute deviations). Sensitivity analyses compared the original global CMC aggregation with analyses excluding flat channels, excluding flat and 50-Hz-screened records, and removing a fitted 50-Hz component after flat-channel exclusion.

**Supplementary Table S2. HD-EMG quality control and CMC sensitivity analysis**

| Metric | Result |
|---|---|
| Audit scope | 80 retained 7-s epochs (40 participants × 2 phases); 2,560 HD-EMG channel epoch records. |
| Data integrity | All records were finite; 18 records (0.70%) had zero variance; 5 records (0.20%) exceeded the descriptive 50-Hz screen. |
| Flat-channel sensitivity | Excluding flat channels: Spearman’s r = 1.000 versus the original global CMC. |
| Line-screen sensitivity | Excluding flat and line-screened channel epochs: Spearman’s r = 0.9998. |
| 50-Hz-removal sensitivity | Removing the fitted 50-Hz component after flat-channel exclusion: Spearman’s r = 0.9977. |
| Inference | No FDR-adjusted C3/C4 comparison was significant for any sensitivity variant. |

**Data availability note:**

The manuscript and this addendum report aggregate quality control counts and sensitivity results. Individual raw EEG/HD-EMG recordings are not included in the manuscript or Supplementary Material and may be considered upon reasonable request in accordance with the study’s data governance requirements.
